# Diaqua­bis[3-(2-hydroxy­ethyl)-2-methyl-4-oxopyrido[1,2-*a*]pyrimidin-9-olato-κ^2^ *N* ^1^,*O* ^9^]manganese(II)

**DOI:** 10.1107/S160053680801369X

**Published:** 2008-05-10

**Authors:** Yu Sun, Xin-Dong Jiang, Xiu-Bing Li

**Affiliations:** a Ordered Matter Science Research Center, College of Chemistry and Chemical Engineering, Southeast University, Nanjing 210096, People’s Republic of China; b College of Pharmacy, Jiangsu University, Zhenjiang 212013, People’s Republic of China

## Abstract

The title compound, [Mn(C_11_H_11_N_2_O_3_)_2_(H_2_O)_2_], consists of discrete mononuclear complex mol­ecules. The Mn^II^ atom is located on an inversion center and coordinated by two N atoms and two O atoms, each pair in a *trans* mode, from two 3-(2-hydroxy­ethyl)-2-methyl-4-oxopyrido[1,2-*a*]pyrimidin-9-olate ligands and by two water mol­ecules. The coordination geometry around the Mn^II^ atom is slightly distorted octa­hedral. Mol­ecules are linked by O—H⋯O hydrogen bonds into a three-dimensional network.

## Related literature

For related literature, see: Bayot *et al.* (2006 ▶); Chen *et al.* (2007 ▶); Wu *et al.* (2006 ▶).
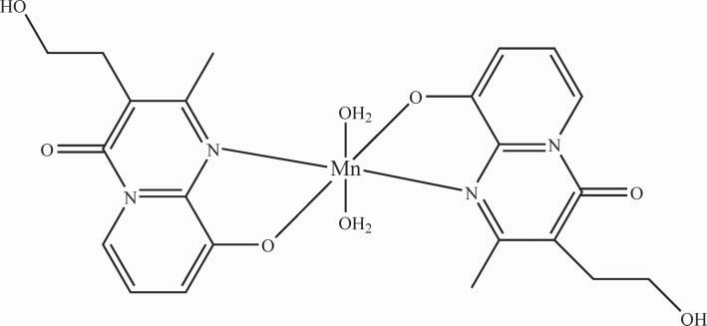


## Experimental

### 

#### Crystal data

[Mn(C_11_H_11_N_2_O_3_)_2_(H_2_O)_2_]
*M* *_r_* = 529.41
Monoclinic, 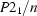

*a* = 5.2656 (11) Å
*b* = 14.620 (3) Å
*c* = 14.715 (3) Å
β = 97.35 (3)°
*V* = 1123.5 (4) Å^3^
*Z* = 2
Mo *K*α radiation
μ = 0.65 mm^−1^
*T* = 293 (2) K
0.15 × 0.12 × 0.06 mm

#### Data collection

Rigaku Scxmini 1K CCD area-detector diffractometer
Absorption correction: multi-scan (*CrystalClear*; Rigaku, 2005 ▶) *T*_min_ = 0.904, *T*_max_ = 0.965
9361 measured reflections
1971 independent reflections
1553 reflections with *I* > 2σ(*I*)
*R*_int_ = 0.063

#### Refinement

*R*[*F* ^2^ > 2σ(*F* ^2^)] = 0.047
*wR*(*F* ^2^) = 0.108
*S* = 1.04
1971 reflections
172 parameters
1 restraint
H atoms treated by a mixture of independent and constrained refinement
Δρ_max_ = 0.27 e Å^−3^
Δρ_min_ = −0.25 e Å^−3^

### 

Data collection: *CrystalClear* (Rigaku, 2005 ▶); cell refinement: *CrystalClear*; data reduction: *CrystalClear*; program(s) used to solve structure: *SHELXS97* (Sheldrick, 2008 ▶); program(s) used to refine structure: *SHELXL97* (Sheldrick, 2008 ▶); molecular graphics: *SHELXTL* (Sheldrick, 2008 ▶); software used to prepare material for publication: *SHELXTL*.

## Supplementary Material

Crystal structure: contains datablocks I, global. DOI: 10.1107/S160053680801369X/hy2131sup1.cif

Structure factors: contains datablocks I. DOI: 10.1107/S160053680801369X/hy2131Isup2.hkl

Additional supplementary materials:  crystallographic information; 3D view; checkCIF report

## Figures and Tables

**Table:** 

Mn1—O1	2.143 (2)
Mn1—O1*W*	2.166 (2)
Mn1—N1	2.368 (2)

**Table:** 

O1^i^—Mn1—O1*W*	92.31 (9)
O1—Mn1—O1*W*	87.69 (9)
O1^i^—Mn1—N1	106.56 (8)
O1—Mn1—N1	73.44 (8)
O1*W*^i^—Mn1—N1	92.98 (9)
O1*W*—Mn1—N1	87.02 (9)

Symmetry code: (i) 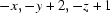.

**Table 2:** Hydrogen-bond geometry (Å, °)

*D*—H⋯*A*	*D*—H	H⋯*A*	*D*⋯*A*	*D*—H⋯*A*
O1*W*—H1*WB*⋯O3^ii^	0.83 (4)	1.94 (5)	2.761 (4)	171 (4)
O1*W*—H1*WA*⋯O1^iii^	0.81 (5)	1.87 (5)	2.680 (3)	177 (5)
O3—H3*B*⋯O2^iv^	0.80 (3)	1.98 (4)	2.772 (4)	168 (4)

Symmetry codes: (ii) 
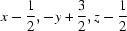
; (iii) 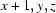; (iv) 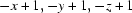.

**Fig. 1.**
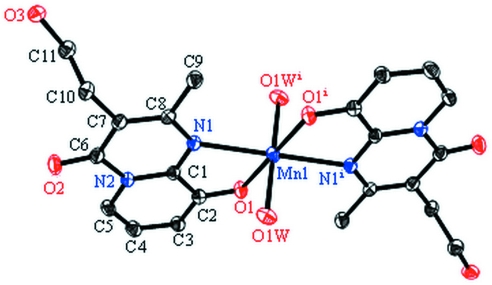
The molecular structure of the title compound. Displacement ellipsoids are drawn at the 30% probability level. H atoms have been omitted for clarity. [Symmetry code: (i) -x, -y + 2, -z + 1.]

**Fig. 2.**
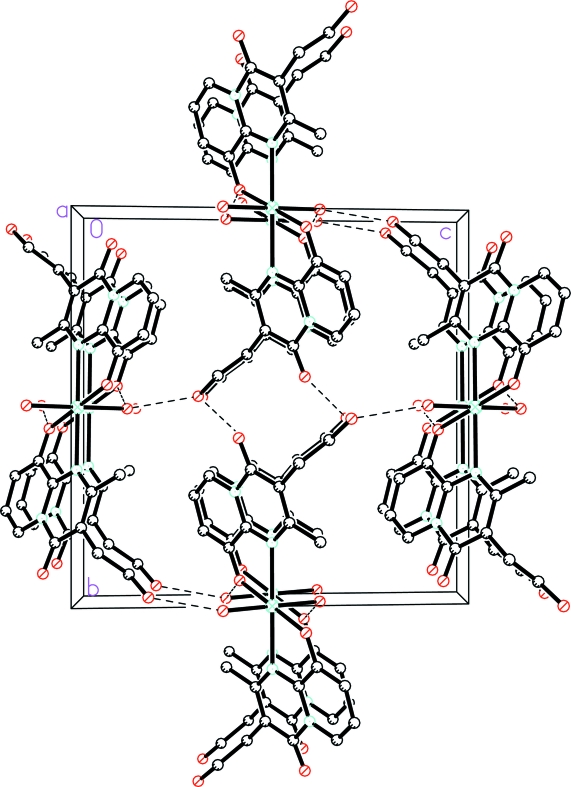
Crystal packing of the title compound. Hydrogen bonds are shown as dashed lines. H atoms have been omitted for clarity.

## supplementary crystallographic information

### Comment

In the past decade, much attention has been paid to the design and synthesis of self-assembling systems with organic ligands containing N and O donors (Bayot *et al.*, 2006; Chen *et al.*, 2007). Quinolin-8-ol is such a ligand and the crystal structure of a complex containing it has been reported (Wu *et al.*, 2006). We report here the synthesis and crystal structure of the title compound.

In the title compound (Fig. 1), the Mn^II^ atom is located on a crystallographic inversion center and adopts a distorted octahedral coordination geometry. The coordination environment is defined by two N atoms and two O atoms from two ligands in the equatorial plane and by two water molecules in the axial positions (Table 1). Intermolecular O—H···O hydrogen bonds involving the hydroxyl groups and water molecules as donors connect the molecules into a three-dimensional network (Table 2; Fig. 2).

### Experimental

Manganese carbonate (0.028 g, 0.1 mmol) was added with constant stirring to a ethanol solution (10 ml) containing 3-(2-hydroxyethyl)-2-methyl-9-hydroxylpyrido[1,2-*a*]pyrimidin-4-one (0.022 g, 0.1 mmol). The mixture was then filtered off. After a few days, brown single crystals in the form of rectangular blocks deposited. They were separated off, washed with cold ethanol and dried in air at room temperature.

### Refinement

H atoms bound to C atoms were positioned geometrically and refined as riding atoms, with C—H = 0.93 (aromatic), 0.97 (CH_2_) and 0.96 Å (CH_3_) and *U*_iso_(H) = 1.2(or 1.5 for methyl)*U*_eq_(C). H atoms of hydroxyl group and water molecule were located in a difference Fourier map and refined isotropically, with a restraint of O—H = 0.82 (1) Å for the hydroxyl group.

### Crystal data

**Table:**

[Mn(C_11_H_11_N_2_O_3_)_2_(H_2_O)_2_]	*F*_000_ = 550
*M**_r_* = 529.41	*D*_x_ = 1.565 Mg m^−^^3^
Monoclinic, *P*2_1_/*n*	Mo *K*α radiation λ = 0.71073 Å
Hall symbol: -P 2yn	Cell parameters from 4792 reflections
*a* = 5.2656 (11) Å	θ = 3.1–25.0º
*b* = 14.620 (3) Å	µ = 0.65 mm^−^^1^
*c* = 14.715 (3) Å	*T* = 293 (2) K
β = 97.35 (3)º	Block, brown
*V* = 1123.5 (4) Å^3^	0.15 × 0.12 × 0.06 mm
*Z* = 2	

### Data collection

**Table:**

Rigaku Scxmini 1K CCD area-detector diffractometer	1971 independent reflections
Radiation source: fine-focus sealed tube	1553 reflections with *I* > 2σ(*I*)
Monochromator: graphite	*R*_int_ = 0.063
Detector resolution: 8.192 pixels mm^-1^	θ_max_ = 25.0º
*T* = 293(2) K	θ_min_ = 3.1º
thin–slice ω scans	*h* = −6→6
Absorption correction: multi-scan(CrystalClear; Rigaku, 2005)	*k* = −17→17
*T*_min_ = 0.904, *T*_max_ = 0.965	*l* = −17→17
9361 measured reflections	

### Refinement

**Table:**

Refinement on *F*^2^	Secondary atom site location: difference Fourier map
Least-squares matrix: full	Hydrogen site location: inferred from neighbouring sites
*R*[*F*^2^ > 2σ(*F*^2^)] = 0.047	H atoms treated by a mixture of independent and constrained refinement
*wR*(*F*^2^) = 0.108	*w* = 1/[σ^2^(*F*_o_^2^) + (0.0407*P*)^2^ + 1.0124*P*] where *P* = (*F*_o_^2^ + 2*F*_c_^2^)/3
*S* = 1.05	(Δ/σ)_max_ < 0.001
1971 reflections	Δρ_max_ = 0.27 e Å^−^^3^
172 parameters	Δρ_min_ = −0.25 e Å^−^^3^
1 restraint	Extinction correction: none
Primary atom site location: structure-invariant direct methods	

### Special details

**Table:**

Geometry. All e.s.d.'s (except the e.s.d. in the dihedral angle between two l.s. planes) are estimated using the full covariance matrix. The cell e.s.d.'s are taken into account individually in the estimation of e.s.d.'s in distances, angles and torsion angles; correlations between e.s.d.'s in cell parameters are only used when they are defined by crystal symmetry. An approximate (isotropic) treatment of cell e.s.d.'s is used for estimating e.s.d.'s involving l.s. planes.

### Fractional atomic coordinates and isotropic or equivalent isotropic displacement parameters (Å^2^)

**Table:**

	*x*	*y*	*z*	*U*_iso_*/*U*_eq_	
Mn1	0.0000	1.0000	0.5000	0.0287 (2)	
N2	−0.0870 (5)	0.71347 (17)	0.40610 (17)	0.0305 (6)	
C8	0.2483 (6)	0.7863 (2)	0.5424 (2)	0.0299 (7)	
C7	0.2868 (6)	0.6962 (2)	0.5179 (2)	0.0331 (7)	
C1	−0.1042 (6)	0.8032 (2)	0.4332 (2)	0.0276 (7)	
C6	0.1200 (6)	0.6554 (2)	0.4466 (2)	0.0349 (8)	
C2	−0.3130 (6)	0.8590 (2)	0.3894 (2)	0.0304 (7)	
C3	−0.4831 (6)	0.8190 (2)	0.3220 (2)	0.0358 (8)	
H3A	−0.6164	0.8534	0.2918	0.043*	
C10	0.4954 (6)	0.6367 (2)	0.5655 (2)	0.0374 (8)	
H10A	0.6367	0.6748	0.5920	0.045*	
H10B	0.5583	0.5962	0.5212	0.045*	
C4	−0.4570 (7)	0.7271 (2)	0.2988 (2)	0.0415 (8)	
H4A	−0.5763	0.7011	0.2541	0.050*	
C9	0.4222 (6)	0.8307 (2)	0.6180 (2)	0.0391 (8)	
H9A	0.3679	0.8926	0.6257	0.059*	
H9B	0.5942	0.8306	0.6029	0.059*	
H9C	0.4162	0.7975	0.6739	0.059*	
C11	0.3977 (7)	0.5808 (2)	0.6400 (2)	0.0424 (9)	
H11A	0.3216	0.6216	0.6810	0.051*	
H11B	0.2645	0.5399	0.6124	0.051*	
N1	0.0601 (4)	0.83949 (17)	0.49945 (17)	0.0287 (6)	
C5	−0.2638 (6)	0.6753 (2)	0.3393 (2)	0.0385 (8)	
H5A	−0.2493	0.6143	0.3226	0.046*	
O1W	0.1917 (5)	1.00410 (18)	0.37828 (18)	0.0403 (6)	
O2	0.1298 (5)	0.57713 (15)	0.41699 (17)	0.0468 (6)	
O1	−0.3258 (4)	0.94368 (14)	0.41643 (15)	0.0343 (5)	
O3	0.5924 (5)	0.52796 (17)	0.69223 (16)	0.0471 (6)	
H1WB	0.144 (7)	0.995 (3)	0.323 (3)	0.048 (11)*	
H1WA	0.339 (9)	0.986 (3)	0.388 (3)	0.077 (16)*	
H3B	0.653 (8)	0.495 (2)	0.657 (2)	0.070 (15)*	

### Atomic displacement parameters (Å^2^)

**Table:**

	*U*^11^	*U*^22^	*U*^33^	*U*^12^	*U*^13^	*U*^23^
Mn1	0.0246 (3)	0.0276 (4)	0.0328 (4)	0.0007 (3)	−0.0004 (3)	−0.0054 (3)
N2	0.0335 (14)	0.0257 (14)	0.0325 (15)	0.0011 (11)	0.0045 (12)	−0.0006 (11)
C8	0.0295 (16)	0.0291 (17)	0.0313 (17)	−0.0005 (14)	0.0046 (13)	0.0042 (13)
C7	0.0350 (18)	0.0299 (18)	0.0349 (18)	0.0028 (14)	0.0063 (14)	0.0035 (14)
C1	0.0286 (16)	0.0254 (16)	0.0301 (16)	−0.0011 (13)	0.0085 (13)	0.0006 (13)
C6	0.0389 (18)	0.0293 (18)	0.0374 (18)	0.0045 (14)	0.0083 (15)	0.0032 (14)
C2	0.0264 (16)	0.0314 (17)	0.0340 (18)	−0.0011 (13)	0.0059 (13)	−0.0006 (14)
C3	0.0325 (17)	0.0357 (18)	0.0368 (19)	−0.0002 (14)	−0.0045 (14)	−0.0035 (14)
C10	0.0375 (18)	0.0300 (18)	0.044 (2)	0.0045 (15)	0.0041 (15)	0.0037 (15)
C4	0.041 (2)	0.042 (2)	0.039 (2)	−0.0059 (16)	−0.0030 (16)	−0.0081 (16)
C9	0.0379 (19)	0.0322 (18)	0.044 (2)	0.0029 (15)	−0.0067 (15)	−0.0009 (15)
C11	0.043 (2)	0.040 (2)	0.043 (2)	0.0061 (16)	0.0035 (16)	0.0033 (16)
N1	0.0252 (14)	0.0262 (14)	0.0332 (14)	−0.0008 (10)	−0.0016 (12)	0.0000 (11)
C5	0.045 (2)	0.0322 (18)	0.0376 (19)	−0.0078 (16)	0.0025 (16)	−0.0091 (15)
O1W	0.0290 (13)	0.0563 (16)	0.0350 (14)	0.0032 (13)	0.0015 (10)	−0.0080 (13)
O2	0.0568 (16)	0.0277 (13)	0.0552 (16)	0.0052 (11)	0.0045 (12)	−0.0095 (11)
O1	0.0263 (11)	0.0286 (12)	0.0456 (13)	0.0031 (9)	−0.0047 (10)	−0.0063 (10)
O3	0.0625 (17)	0.0437 (15)	0.0330 (14)	0.0158 (13)	−0.0024 (12)	0.0027 (11)

### Geometric parameters (Å, °)

**Table:**

Mn1—O1^i^	2.143 (2)	C2—C3	1.378 (4)
Mn1—O1	2.143 (2)	C3—C4	1.398 (5)
Mn1—O1W^i^	2.166 (2)	C3—H3A	0.9300
Mn1—O1W	2.166 (2)	C10—C11	1.510 (5)
Mn1—N1^i^	2.368 (2)	C10—H10A	0.9700
Mn1—N1	2.368 (2)	C10—H10B	0.9700
N2—C1	1.378 (4)	C4—C5	1.346 (5)
N2—C5	1.382 (4)	C4—H4A	0.9300
N2—C6	1.448 (4)	C9—H9A	0.9600
C8—N1	1.351 (4)	C9—H9B	0.9600
C8—C7	1.387 (4)	C9—H9C	0.9600
C8—C9	1.495 (4)	C11—O3	1.425 (4)
C7—C6	1.411 (5)	C11—H11A	0.9700
C7—C10	1.502 (4)	C11—H11B	0.9700
C1—N1	1.328 (4)	C5—H5A	0.9300
C1—C2	1.453 (4)	O1W—H1WB	0.81 (4)
C6—O2	1.228 (4)	O1W—H1WA	0.82 (5)
C2—O1	1.304 (4)	O3—H3B	0.80 (3)
			
O1^i^—Mn1—O1	180.0	C2—C3—H3A	119.7
O1^i^—Mn1—O1W^i^	87.69 (9)	C4—C3—H3A	119.7
O1—Mn1—O1W^i^	92.31 (9)	C7—C10—C11	110.8 (3)
O1^i^—Mn1—O1W	92.31 (9)	C7—C10—H10A	109.5
O1—Mn1—O1W	87.69 (9)	C11—C10—H10A	109.5
O1W^i^—Mn1—O1W	180.000 (1)	C7—C10—H10B	109.5
O1^i^—Mn1—N1	106.56 (8)	C11—C10—H10B	109.5
O1—Mn1—N1	73.44 (8)	H10A—C10—H10B	108.1
O1W^i^—Mn1—N1	92.98 (9)	C5—C4—C3	121.7 (3)
O1W—Mn1—N1	87.02 (9)	C5—C4—H4A	119.2
O1^i^—Mn1—N1^i^	73.44 (8)	C3—C4—H4A	119.2
O1—Mn1—N1^i^	106.56 (8)	C8—C9—H9A	109.5
O1W^i^—Mn1—N1^i^	87.02 (9)	C8—C9—H9B	109.5
O1W—Mn1—N1^i^	92.98 (9)	H9A—C9—H9B	109.5
N1—Mn1—N1^i^	180.000 (1)	C8—C9—H9C	109.5
C1—N2—C5	121.9 (3)	H9A—C9—H9C	109.5
C1—N2—C6	120.8 (3)	H9B—C9—H9C	109.5
C5—N2—C6	117.3 (3)	O3—C11—C10	113.4 (3)
N1—C8—C7	123.1 (3)	O3—C11—H11A	108.9
N1—C8—C9	116.2 (3)	C10—C11—H11A	108.9
C7—C8—C9	120.7 (3)	O3—C11—H11B	108.9
C8—C7—C6	119.8 (3)	C10—C11—H11B	108.9
C8—C7—C10	123.4 (3)	H11A—C11—H11B	107.7
C6—C7—C10	116.8 (3)	C1—N1—C8	118.9 (3)
N1—C1—N2	122.2 (3)	C1—N1—Mn1	108.89 (18)
N1—C1—C2	119.1 (3)	C8—N1—Mn1	131.21 (19)
N2—C1—C2	118.7 (3)	C4—C5—N2	119.3 (3)
O2—C6—C7	127.4 (3)	C4—C5—H5A	120.3
O2—C6—N2	117.6 (3)	N2—C5—H5A	120.3
C7—C6—N2	115.0 (3)	Mn1—O1W—H1WB	134 (3)
O1—C2—C3	124.6 (3)	Mn1—O1W—H1WA	113 (3)
O1—C2—C1	117.6 (3)	H1WB—O1W—H1WA	106 (4)
C3—C2—C1	117.8 (3)	C2—O1—Mn1	118.08 (18)
C2—C3—C4	120.6 (3)	C11—O3—H3B	107 (3)
			
N1—C8—C7—C6	−2.0 (5)	C7—C10—C11—O3	−175.6 (3)
C9—C8—C7—C6	179.8 (3)	N2—C1—N1—C8	−0.9 (4)
N1—C8—C7—C10	−179.9 (3)	C2—C1—N1—C8	177.5 (3)
C9—C8—C7—C10	1.8 (5)	N2—C1—N1—Mn1	168.9 (2)
C5—N2—C1—N1	177.7 (3)	C2—C1—N1—Mn1	−12.6 (3)
C6—N2—C1—N1	−2.6 (4)	C7—C8—N1—C1	3.3 (4)
C5—N2—C1—C2	−0.7 (4)	C9—C8—N1—C1	−178.4 (3)
C6—N2—C1—C2	179.0 (3)	C7—C8—N1—Mn1	−163.9 (2)
C8—C7—C6—O2	179.6 (3)	C9—C8—N1—Mn1	14.4 (4)
C10—C7—C6—O2	−2.3 (5)	O1^i^—Mn1—N1—C1	−166.19 (19)
C8—C7—C6—N2	−1.4 (4)	O1—Mn1—N1—C1	13.81 (19)
C10—C7—C6—N2	176.6 (3)	O1W^i^—Mn1—N1—C1	105.3 (2)
C1—N2—C6—O2	−177.3 (3)	O1^i^—Mn1—N1—C8	2.0 (3)
C5—N2—C6—O2	2.4 (4)	O1—Mn1—N1—C8	−178.0 (3)
C1—N2—C6—C7	3.6 (4)	O1W^i^—Mn1—N1—C8	−86.5 (3)
C5—N2—C6—C7	−176.7 (3)	O1W—Mn1—N1—C8	93.5 (3)
N1—C1—C2—O1	1.3 (4)	C3—C4—C5—N2	0.4 (5)
N2—C1—C2—O1	179.8 (3)	C1—N2—C5—C4	0.7 (5)
N1—C1—C2—C3	−178.9 (3)	C6—N2—C5—C4	−179.0 (3)
N2—C1—C2—C3	−0.4 (4)	C3—C2—O1—Mn1	−166.9 (2)
O1—C2—C3—C4	−178.8 (3)	C1—C2—O1—Mn1	12.9 (3)
C1—C2—C3—C4	1.4 (5)	O1W^i^—Mn1—O1—C2	−106.8 (2)
C8—C7—C10—C11	94.0 (4)	O1W—Mn1—O1—C2	73.2 (2)
C6—C7—C10—C11	−84.0 (4)	N1—Mn1—O1—C2	−14.4 (2)
C2—C3—C4—C5	−1.5 (5)	N1^i^—Mn1—O1—C2	165.6 (2)

Symmetry codes: (i) −*x*, −*y*+2, −*z*+1.

### Hydrogen-bond geometry (Å, °)

**Table:**

*D*—H···*A*	*D*—H	H···*A*	*D*···*A*	*D*—H···*A*
O1W—H1WB···O3^ii^	0.83 (4)	1.94 (5)	2.761 (4)	171 (4)
O1W—H1WA···O1^iii^	0.81 (5)	1.87 (5)	2.680 (3)	177 (5)
O3—H3B···O2^iv^	0.80 (3)	1.98 (4)	2.772 (4)	168 (4)

Symmetry codes: (ii) *x*−1/2, −*y*+3/2, *z*−1/2; (iii) *x*+1, *y*, *z*; (iv) −*x*+1, −*y*+1, −*z*+1.
